# Description and Analysis of Cytokine Storm in Registered COVID-19 Clinical Trials: A Systematic Review

**DOI:** 10.3390/pathogens10060692

**Published:** 2021-06-02

**Authors:** Khalid Eljaaly, Husam Malibary, Shaimaa Alsulami, Muradi Albanji, Mazen Badawi, Jaffar A. Al-Tawfiq

**Affiliations:** 1 Faculty of Pharmacy, Department of Pharmacy Practice, King Abdulaziz University, Jeddah 21589, Saudi Arabia; 2 College of Pharmacy, Department of Pharmacy Practice and Science, King Abdulaziz University, Tucson, AZ 85724, USA; 3 Allergy and Clinical Immunology Division, Faculty of Medicine, Department of Internal Medicine, King Abdulaziz University, Jeddah 21589, Saudi Arabia; hmmalibary@kau.edu.sa; 4 Department of Pharmacy, King Abdulaziz University Hospital, Jeddah 21589, Saudi Arabia; alsulamish3@nhga.med.sa (S.A.); Albanji@kau.edu.sa (M.A.); 5 Department of Pharmacy, King Fahad Armed Forces Hospital, Jeddah 21159, Saudi Arabia; 6 Infectious Disease Division, Faculty of Medicine, Department of Internal Medicine, King Abdulaziz University, Jeddah 21589, Saudi Arabia; mabadawi@kau.edu.sa; 7 Infectious Disease Unit, Specialty Internal Medicine, Johns Hopkins Aramco Healthcare, Dhahran 31311, Saudi Arabia; jaffar.tawfiq@jhah.com; 8 Infectious Disease Division, Department of Medicine, Indiana University School of Medicine, Indianapolis, IN 46202, USA; 9 Infectious Disease Division, Department of Medicine, Johns Hopkins University, Baltimore, MD 21287, USA

**Keywords:** cytokine storm, cytokine release syndrome, definition, systematic review

## Abstract

The purpose of this systematic review was to describe the characteristics of clinical trials that focused on COVID-19 patients with cytokine release syndrome (CRS) and the variability in CRS definitions. Two authors independently searched three clinical trial registries and included interventional clinical trials on COVID-19 hospitalized patients that required at least one elevated inflammatory biomarker. Relevant data, including the type and cutoff of the measured biomarker, oxygen/respiratory criteria, fever, radiologic criteria, and medications, were summarized. A total of 47 clinical trials were included. The included studies considered the following criteria: oxygen/respiratory criteria in 42 trials (89%), radiologic criteria in 29 trials (62%), and fever in 6 trials (18%). Serum ferritin was measured in 35 trials (74%), CRP in 34 trials (72%), D-dimer in 26 trials (55%), LDH in 24 trials (51%), lymphocyte count in 14 trials (30%), and IL-6 in 8 trials (17%). The cutoff values were variable for the included biomarkers. The most commonly used medications were tocilizumab, in 15 trials (32%), and anakinra in 10 trials (24.4%). This systematic review found high variability in CRS definitions and associated biomarker cutoff values in COVID-19 clinical trials. We call for a standardized definition of CRS, especially in COVID-19 patients.

## 1. Introduction

Coronavirus disease 2019 (COVID-19) has rapidly spread across continents and became a global pandemic [1]. Patients with COVID-19 frequently experience pneumonia and may develop acute respiratory distress syndrome (ARDS). ARDS is characterized by bilateral lung infiltrates and severe progressive hypoxemia, and patients need to be admitted to the critical care unit and receive respiratory support [2,3]. ARDS is a rapidly progressive condition and is associated with a high mortality rate in COVID-19 patients. Severe acute respiratory syndrome coronavirus 2 (SARS-CoV-2) seems to cause comparable immunopathogenic features as those seen in SARS-CoV and MERS-CoV infections [2]. ARDS is one of the features of cytokine release syndrome (CRS), also known as cytokine storm, and its exact mechanism is still not well understood [4]. Hyperactive immune responses and cytokine overproduction have been associated with the pathogenesis of infectious and non-infectious diseases [4]. The term CRS captured the attention not only of the scientific publications but also of the media. CRS is a systemic inflammatory response mediated by the overproduction of pro-inflammatory cytokines, which can be stimulated by different factors, including infections and certain medications [5,6]. CRS is a major cause of morbidity in patients infected with SARS-CoV and MERS-CoV [7]. CRS was first described as an adverse reaction of the solid organ immunosuppressive medication, an anti-T cell antibody muromonab-CD 3 (OKT3), in the early 1990s [8]. Although the incidence of CRS with conventional monoclonal antibodies is relatively low, it is relatively high with T cell-engaging cancer immunotherapy [9]. Several clinical factors are associated with the severity of CRS following chimeric antigen receptor (CAR) T cell therapy [10]. Its severity ranges from mild symptoms, such as flu-like symptoms, fever, fatigue, headache, rash, arthralgia, and myalgia, to severe life-threatening reactions characterized by hypotension and high-grade fever. CRS can also progress to an uncontrolled systemic inflammatory response with vasopressor-requiring circulatory shock, vascular leakage, disseminated intravascular coagulation, and multi-organ system failure. CRS respiratory symptoms are common and can be mild as cough and tachypnea. However, it may progress to ARDS with dyspnea, hypoxia, and bilateral opacities on a chest x-ray requiring mechanical ventilation. Life-threatening CRS associated with mechanical ventilation is not caused by the respiratory disease mechanism alone but also the inability to protect the airway due to secondary neurotoxicity [11]. Multi-organ dysfunction in severe CRS includes renal failure, cardiac dysfunction with reduced ejection fraction, and vascular leakage with peripheral and pulmonary edema. Laboratory abnormalities associated with CRS include high inflammatory biomarkers. Severe CRS might be associated with laboratory abnormalities resembling hemophagocytic lymphohistiocytosis (HLH) or macrophage activation syndrome (MAS) [12]. It has been suggested that severe COVID-19 patients should be screened for hyperinflammation by using laboratory biomarkers and HScore. Hscore is usually used to generate a probability for the presence of secondary HLH, and to recognize the subgroup of patients for whom immunosuppression could decrease ARDS and mortality [13]. In depth understanding of the clinical picture and the underlining pathophysiology of CRS is crucial to establish early and effective management of this syndrome. There is no consensus on the definition of CRS and associated changes in inflammatory biomarkers [4,6]. Therefore, the objective of this systematic review was to describe the variability in identifying patients with cytokine storm in clinical trials of COVID-19 patients and the relevant characteristics of these trials.

## 2. Materials and Methods

Two investigators independently screened the ClinicalTrials.gov, European Union Clinical Trials Register, and World Health Organization International Clinical Trials Registry Platform between 1 November 2019 and 23 October 2020. The search strategy is provided in Table 1. We included interventional clinical trials on COVID-19 hospitalized patients that required at least one elevated inflammatory biomarker (CRP, ferritin, D-dimer, LDH, IL-6, or lymphocyte count) in their inclusion criteria and mentioned any of the following terminologies “cytokine storm”, “cytokine storm syndrome”, “cytokine release syndrome”, “hyperinflammation”, “macrophage activation syndrome”, “immune dysregulation”, or “hemophagocytic lymphohistiocytosis”. From each clinical trial, the following data were extracted: registration number, recruitment country, whether included patients were adults or pediatrics, studied medications, the type and cutoff of measured biomarkers, whether fever, oxygen/respiratory, and/or radiologic criteria were used in addition to the biomarkers. The unit of each biomarker was converted to the one most commonly used unit for convenience to allow for numerical comparison (µg/L for ferritin, mg/L for CRP, ng/mL for D-dimer, cells/µL for lymphocytes, IU/L for LDH, and pg/mL for IL-6).

## 3. Results

**Figure 1:**
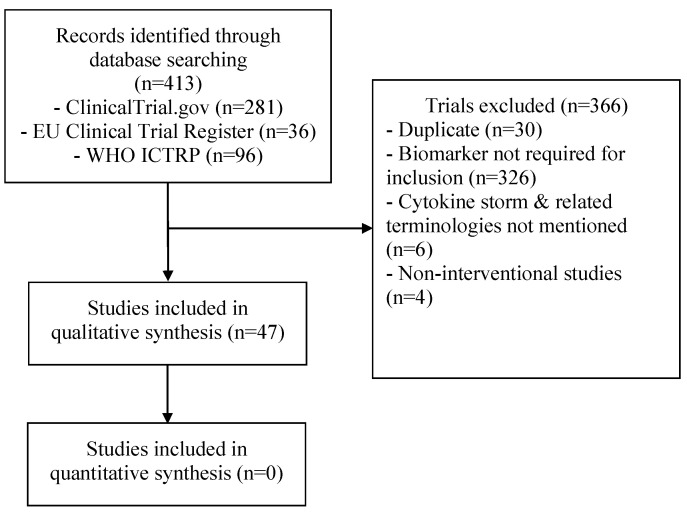
Flow diagram of the study selection process.

Out of 413 clinical trials screened, a total of 47 trials were included (Figure 1). The study characteristics of these studies are presented in Table 2 and Table A1, and a summary of the included parameters in the definition of the different studies is provided in Table 3. A total of 26 trials (55.3%) were in Europe, 11 (23.4%) were in North America, 2 (4.2%) were in South America, 6 (12.8%) were in Asia, and 2 (4.2%) were multicontinental. Almost all clinical trials included only adult patients and one study included patients ≥12 years, in the United States only (NCT04362813). The most commonly studied medications were as follows: tocilizumab (15 trials; 32%), anakinra (10 trials; 24.4%), corticosteroids (4 trials; 8.5%), sarilumab (4 trials; 8.5%), clazakizumab (3 trials; 6.3%), ruxolitinib (3 trials; 6.3%), emapalumab (2 trials; 4.2%), and siltuximab (2 trials; 4.2%). The following interventions were used in one study each: canakinumab, gimsilumab, itolizumab, mavrilimumab, cytokine adsorption, CytoSorb, hyperbaric oxygen, radiotherapy, collagen-polyvinylpyrrolidone, therapeutic plasma exchange, trimethoprim-sulfamethoxazole, reparixin, losmapimod, defibrotide, dornase alfa, etoposide, pyridostigmine, and zilucoplan. The inclusion criteria that were used in these studies were as follows: oxygen/respiratory criteria in 42 trials (89%), radiologic criteria in 27 trials (62%), and fever in 6 trials (19%). Other used clinical criteria were mainly shock and organ dysfunction in four trials (NCT04424056, NCT04339712, DRKS00021447, NCT04366232). Only one trial included inflammatory biomarkers without clinical or radiological criteria (NCT04423042).

A serum ferritin measurement was required in 35 trials (74%), CRP in 34 trials (72%), D-dimer in 26 trials (55%), LDH in 24 trials (51%), lymphocyte count in 14 trials (30%), and IL-6 in 8 trials (17%). There were 11 studies that included other biomarkers (platelets, WBCs, transaminases, fibrinogen, cross-linked fibrin degradation products, neutrophil-lymphocyte ratio, troponin, creatinine kinase, triglycerides, and hemoglobin). The most common cutoff values for ferritin were >500 µg/L (11 studies), >300 µg/L (6 studies), >1000 µg/L (6 studies), and >2000 µg/L (5 studies). For CRP, the most common cutoff values were >30–35 mg/L (7 studies), >70 mg/L (6 studies), >50 mg/L (5 studies), and >100 mg/L (3 studies). The most common D-dimer cutoff values were >1000 ng/mL (18 studies), >500 ng/mL (3 studies), and >1500 ng/mL (3 studies). For lymphocyte counts, the most common cutoff values were <500 cells/µL (5 studies) and <1000 cells/µL (5 studies). The most common LDH cutoff values were >300 IU/L (7 studies), >245–250 IU/L (4 studies), and >200 IU/L (3 studies). For IL-6, the most common cutoff value was >40 pg/mL (3 studies).

## 4. Discussion

CRS is a systemic inflammatory response caused by the release of inflammatory cytokines, such as IL-6, interferon gamma (IFNγ), tumor necrosis factor-alpha (TNFα), IL-2, and IL-10. This is the result of the activation of a large number of lymphocytes (B cells, T cells, and/or natural killer cells) and/or myeloid cells (macrophages, dendritic cells, and monocytes). CRS can manifest with a constellation of clinical symptoms including fever, hypotension, and widespread organ dysfunction [14]. There is no international consensus on unified criteria for the diagnosis of CRS. In addition, cytokine elevation was thought to be a late finding in patients with COVID-19 [15].

Although CRS is increasingly described in patients with severe or critical COVID-19, our review demonstrates that there is significant variability in the definition and defining criteria for CRS in the included prospective trials. Most of the included studies involved patients who meet the severe to critical case definition of COVID-19, as per the American National Institutes of Health (NIH) [1]. This finding implies that CRS occurrence is limited to those with severe and critical cases. Several grading scales for the severity of CRS have been suggested. Originally, CAR T cell therapy-induced CRS was categorized according to the Common Terminology Criteria for Adverse Events version 4.03 (CTCAEv4.03), released in 2009. This grading scale was used to classify CRS-related adverse events caused by immunotherapies [16]. Since then, several attempts were made to develop a more concise grading scale. In 2014, Lee et al. modified the CRS grading, as per CTCAEv4.03, to define mild, moderate, severe, and life-threatening CRS regardless of the inciting agent [14]. Another published rating scale for CRS was devised by Davila et al., in 2015 [17]. In 2018, Neelapu et al. proposed a grading scale for CRS very similar to the Lee criteria [18]. In March 2018, CTCAE v5.0 was published with significantly modified grading criteria for CRS that account for patients’ responses to fluids, oxygen requirements, need for vasopressors, and organ dysfunction [19]. Recently, the American Society for Blood and Marrow Transplantation sponsored a meeting to come to a consensus for CRS and CAR-associated neurotoxicity grading [20]. In the present review, all but one (46/47) of the prospective trials included clinical manifestations in their inclusion criteria, with hypoxia being the most commonly included. This finding is in contrast to what was found in the above-mentioned grading scales of CRS, where fever and hypotension were the most common included criteria. It is important to realize that most of the CRS cases examined during the development of these grading scales were secondary to CAR T cell and other immunotherapies, which may provoke more profound cytokine release and systemic inflammation. In our review, the CRS patients included in these trials are COVID-19 patients, and as such, hypoxia may be attributed to viral pneumonia rather than the development of CRS per se. Regarding the radiographic investigations, 29 studies out of 47 (62%) mandated the presence of radiologic changes in chest X-rays or CT scans and thus, the contribution of radiological changes to the diagnosis of CRS is not a unified criterion for the diagnosis of CRS. Radiographic features of ARDS are the most commonly described changes in CRS. However, the sensitivity and specificity of these changes are not known yet.

We could not elucidate any specific biochemical biomarker for an accurate diagnosis of CRS. All of the included studies mandated the presence of laboratory abnormality. There are several inflammatory markers to be considered with this condition, including CRP, ferritin, D-dimer, LDH, IL-6, and lymphocyte count. However, serum ferritin and CRP are the most selected biomarkers (74% and 72%, respectively). The sensitivity and specificity of these biomarkers for CRS are yet to be evaluated. Other biomarkers included in these trials were fibrinogen, cross-linked fibrin degradation products, platelets, hemoglobin, WBCs, creatinine kinase, triglycerides, liver enzymes, and troponin. As mentioned above, CRS is associated with multi-organ involvement and hence, many biochemical abnormalities might be present. Moreover, it seems that there are no clear diagnostic cutoffs for these biomarkers in CRS. We found significant variations in the cutoff levels of these biomarkers among the included studies. Davila et al. added cytokine elevation as a criterion for severe CRS, defined as at least a 75-fold elevation of two serum cytokines over baseline, or a 250-fold elevation of at least one serum cytokine over baseline [17]. Additionally, they identified 7 cytokines, of the 39 measured, whose elevation strongly correlated with CRS. These cytokines were IFN-γ, IL5, IL6, IL10, Flt-3L, Fractalkine, and GM-CSF [17].

In a recent rapid systematic review, Leisman DE et al. compared the maximum levels of several inflammatory biomarkers (TNFα, IL-8, IL-1β, IL-10, IL-2, IL-4, soluble IL-2 receptor, IFNγ, CRP, ferritin, fibrinogen, D-dimer, LDH, ESR, albumin, procalcitonin, total bilirubin, lymphocyte count, and platelet count) between COVID-19-induced CRS and three other systemic inflammatory conditions: sepsis, non-COVID-19-related ARDS, and non-COVID-19-induced CRS [21]. The calculated mean IL-6 level in COVID-19 patients was 36.7 pg/mL (ranging between 6.5 and 357.2 pg/mL). The mean IL-6 serum level was nearly 100 times higher in patients with CAR T cell-induced CRS compared with patients with COVID-19. Similarly, the pooled mean IL-6 level was much higher in patients with hyperinflammatory ARDS (1558.2 pg/ mL) and patients with sepsis (983.6 pg/mL) compared with COVID-19 patients. Patients with COVID-19 had substantially higher D-dimer elevations than did patients with sepsis. Most other cytokines were comparatively low in COVID-19 patients [21]. These data may explain why anti-cytokine therapy has not been universally effective for the treatment of severe COVID-19 in several randomized clinical trials [1]. However, larger-scale trials are warranted to further explore the role of anti-cytokine therapy and immunomodulators in severe COVID-19 infection, considering their efficacy, safety, and patient characteristics [22,23]. In our systematic review, most trials used monoclonal antibodies, with tocilizumab, followed by anakinra, being the most studied.

It is believed that CRS is a major contributor to increased morbidity and mortality in COVID-19 patients [2]. Currently, the options to control this disease and its complications are quite limited. The use of immune modulators in COVID-19-associated CRS shows some promising results [24]. With a better understanding of CRS and its pathophysiological aspects in COVID-19 infection, more focused efforts can take place to find the proper treatment of COVID-19 patients. This understanding will also help to guide the timing of immune modulator therapy administration, its dosing, and if repeat courses are indicated.

## 5. Conclusions

There is high variability in CRS definitions and associated biomarker cutoff values in the COVID-19 clinical trials. We call to form a unified definition of CRS, especially in COVID-19 patients, as this is a critical step to move forward in designing COVID-19 therapeutics. Such a unified definition would be an important contribution to the scientific community. However, having an expert consensus on such a definition is beyond the scope of this review and deserves international collaborative future work.

## Figures and Tables

**Table 1:** Search Strategy.

Database	Search Strategy
ClinicalTrials.gov	COVID-19 AND (“cytokine” OR “hyperinflammation” OR “macrophage activation syndrome” OR “immune dysregulation” OR “hemophagocytic lymphohistiocytosis”). Restricted to interventional studies (clinical trials).
EU Clinical Trial Register	COVID-19 AND (“cytokine” OR “hyperinflammation” OR “macrophage activation syndrome” OR “immune dysregulation” OR “hemophagocytic lymphohistiocytosis”).
WHO International Clinical Trials Registry Platform	COVID-19 AND cytokine OR COVID-19 AND hyperinflammation OR COVID-19 AND macrophage activation syndrome OR COVID-19 AND immune dysregulation OR COVID-19 AND hemophagocytic lymphohistiocytosis.

**Table 2:** Summary and characteristics of the included studies describing cytokine storm in COVID-19 patients.

Study ID Number	Database	Intervention	Country	CRP (mg/L)	Ferritin (µg/L)	D-dimer (ng/mL)	LDH (IU/L)	Lymphocyte (cells/µL)	IL-6 (pg/mL)	Other Biomarkers	Fever
NCT04443881	CT.gov, WHO ICTRP	Anakinra	Spain	No	>500	No	>300	No	>40	No	No
NCT04356937	CT.gov	Tocilizumab	United States	>50	>500	>1000	>250	No	No	No	Yes
NCT04361526	CT.gov, WHO ICTR	Cytokine Adsorption	Spain	>10	No	No	No	No	No	No	No
NCT04335071	CT.gov	Tocilizumab	Switzerland	≥50	No	No	No	No	No	No	No
2020-001500-41; EUCTR2020-001500-41-BE	EU CTR, WHO ICTRP	Tocilizumab, siltuximab, anakinra	Belgium	>70 and rising since last 24 h	>1000 and rising since last 24 h >2000 in patients requiring immediate high flow oxygen device or mechanical ventilation if lymphopenia and additional criteria >700 and rising since last 24 h	>1000 and rising since last 24 h	>300	<800	No	No	No
NCT04394182	CT.gov, WHO ICTRP	Radiotherapy	Spain	Above normal range	Above the normal range	Above normal range	Above normal range	Below normal range	Above normal range	Fibrinogen	No
NCT04366232; 2020-001963-10	CT.gov; EU CTR	Ruxolitinib, anakinra	France	>150	>5000	No	No	No	No	No	No
NCT04357860	CT.gov, WHO ICTRP	Sarilumab	Spain	No	No	>1500 or >1000 if progressive increases are documented	No	No	>40	No	No
NCT04356690	CT.gov	Etoposide	United States	>100	>1000 or >500 with an additional biomarker	>1000	> 500	No	No	WBC	No
NCT04348383	CT.gov, WHO ICTRP	Defibrotide	Spain	No	No	No	No	No	≥3 × upper normal limit	No	No
NCT04345445	CT.gov, WHO ICTRP	Tocilizumab, methylprednisolone	Malaysia	>60 or an increase >20 over 12 h	Increasing	No	No	Declining	No	No	No
2020-001255-40; EUCTR2020-001255-40-ES	EU CTR, WHO ICTRP	Sarilumab	Spain	>100 or increasing over 24 h	>300	>1500 or progressive increase (over 3 consecutive measurements) and reaching ≥1000	No	< 800	No	No	No
2020-001375-32	EU CTR	Tocilizumab	Netherlands	No	>2000 or doubling in 20–48 h	No	No	No	No	No	No
NCT04403685	CT.gov, EU CTR	Tocilizumab	Brazil	>50	>300	>1000	>upper level limit	No	No	No	No
RPCEC00000311	WHO ICTRP	Itolizumab	Cuba	No	Increased initial value from 500 or absolute value ≥ 2000.	Increase	No	No	No	Hemoglobin, platelets, neutrophils, ESR in mismatch with CRP, triglycerides, ALT, Fibrinogen	Yes
NCT04322773	CT.gov	Tocilizumab, sarilumab	Denmark	>70 or ≥40 and doubled within 48 h	300	>1000	>250	<600	No	Platelet	No
NCT04362111	CT.gov, ET CTR	Anakinra	United States	No	>700	>500	>2 × upper normal limit	<1000	No	WBC, platelet, AST or ALT	Yes
NCT04423042	CT.gov, WHO ICTRP	Tocilizumab	Canada	≥70	>700 and/or rising since last 24 h	No	No	No	No	No	No
ChiCTR2000030196	WHO ICTRP	Tocilizumab	China	No	No	No	No	No	Elevated	No	No
NCT04339712; 2020-001039-29	CT.gov, EU CTR, WHO ICTRP	Tocilizumab, anakinra	Greece	No	>4420	No	No	No	No	No	No
DRKS00021447	WHO ICTRP	CytoSorb	Germany	>100	No	No	No	No	No	No	No
NCT04343963	CT.gov	Pyridostigmine	Mexico	>30	>300	>1000	>245	<800	No	Creatinine Kinase	No
2020-001390-76	EU CTR	Sarilumab	Italy	>30	>500	>1000	>300	<1000	No	No	No
NCT04377503	CT.gov, WHO ICTRP	Tocilizumab, methylprednisolone	Brazil	>50	>300	>1500	>245	No	>7	No	No
NCT04327505; 2020-001349-37	CT.gov, EU CTR, WHO ICTRP	Hyperbaric oxygen	Germany, Sweden	No	No	>1000	No	No	No	No	No
NCT04359654	CT.gov, WHO ICTRP	Dornase alfa inhalation	United Kingdom	≥30	No	No	No	No	No	No	No
NCT04397497	CT.gov	Mavrilimumab	Italy	≥60	≥1000	No	Above normal range	No	No	No	Yes
NCT04424056	CT.gov	Tocilizumab, anakinra, ruxolitinib	France	>150	>5000	No	No	No	No	No	No
NCT04382755	CT.gov	Zilucoplan	Belgium	>70 and rising since last 24 h	>1000 and rising since last 24 h >2000 in patients requiring Optiflow or mechanical ventilation >700 ug/L and rising since last 24 h if lymphopenia and additional criteria	>1000 and rising since last 24 h	>300	<800	No	No	No
NCT04330638	CT.gov, WHO ICTRP	Tocilizumab, anakinra, siltuximab	Belgium	>70 and rising since last 24 h	>1000 and rising since last 24 h >2000 in patients requiring Optiflow or mechanical ventilation >700 ug/L and rising since last 24 h if lymphopenia and additional criteria	>1000 and rising since last 24 h	>300 and rising last 24 h	<800	No	No	No
NCT04324021; 2020-001167-93	CT.gov, EU CTR, WHO ICTRP	Emapalumab, anakinra	Italy	No	>500	>1000	>300	<1000	No	No	No
NCT04381052	CT.gov	Clazakizumab	United States	>35	>500	>1000	>200	No	No	Troponin neutrophil-lymphocyte ratio	No
NCT04343989	CT.gov	Clazakizumab	United States	>35	>500	>1000	>200	No	No	Troponin neutrophil-lymphocyte ratio	No
NCT04363502	CT.gov	Clazakizumab	United States	>35	>500	>1000	>200	No	No	Troponin neutrophil-lymphocyte ratio	No
NCT04359290	CT.gov	Ruxolitinib	Germany	No	Above normal value	No	>283	No	No	No	No
NCT04362813; 2020-001370-30	CT.gov, EU CTR, WHO ICTRP	Canakinumab	United States, France, Germany, Italy, Russia, Spain, United Kingdom	≥20	≥600	No	No	No	No	No	No
NCT04351243	CT.gov	Gimsilumab	United States	Elevated	Elevated	No	No	No	No	No	No
NCT04517162	CT.gov, WHO ICTRP	Collagen-polyvinylpyrrolidone	United States	No	>300	>1000	No	<800	No	Creatinine kinase, troponin	No
NCT04470531	CT.gov	Co-trimoxazole	Bangladesh	>50	No	No	No	No	No	No	No
NCT04560205	CT.gov	Tocilizumab	Pakistan	>50	>1000	>1000	>1000	No	No	No	No
NCT04559113	CT.gov	Methylprednisolone	Pakistan	>20	>500	>500	>600	No	No	No	No
NCT04528888	CT.gov	Methylprednisolone	Italy	>6 × upper normal limit	No	>6 × upper limit of normal	No	No	No	No	No
NCT04457349	CT.gov, WHO ICTRP	Therapeutic Plasma Exchange	Egypt	Persistent high	No	No	No	No	Persistent high	No	Yes
2020-001645-40	EU CTR	Reparixin	Italy	≥30	≥900	No	Elevated	No	≥40	Cross-linked fibrin degradation products	No
2020-001748-24	EU CTR	Tocilizumab, anakinra	Sweden	>70	>500	>500	>470	<1000	No	No	No
NCT04324021; 2020-001167-93	CT.gov, EU CTR, WHO ICTRP	Emapalumab, anakinra	Italy	No	>500	>1000	>300	<1000	No	No	No
NCT04511819	CT.gov	Losmapimod	United States, Brazil, Mexico	>15	No	No	No	No	No	No	No

CT.gov: CliniclTrials.gov; EU CTR: European Union Clinical Trials registry; WHO ICTRP: World Health Organization International Clinical Trials Registry Platform.

**Table 3:** Summary of specified criteria that were used in the different included studies.

Criteria	CRP (mg/L)	Ferritin (µg/L)	D-dimer (ng/mL)	LDH (IU/L)	Lymphocyte (cells/µL)	IL-6 (pg/mL)	Other Biomarkers	Fever	O_2_/Respiratory Criteria	Radiologic Criteria
Number of studies (%)	34 (72%)	35 (74%)	26 (55%)	24 (51%)	14 (30%)	8 (17%)	9 (23%)	6 (18%)	34 (89%)	29 (62%)

## Appendix A

**Table A1:** O_2_/respiratory criteria and radiologic criteria in the included studies describing cytokine storm in COVID-19 patients.

Study ID Number	O_2_/Respiratory Criteria	Radiologic Criteria
NCT04443881	SpO2 ≤ 94% measured with a pulse oximeter Pa:FiO2 ≤ 300 Sa:FiO2 ≤ 350	CXR (or other technique) pulmonary infiltrates compatible with pneumonia
NCT04356937	O2 supplementation not > 10 L delivered by any device Need for supplemental O2 to maintain saturation > 92%	Pulmonary infiltrate on CXR
NCT04361526	Worsening respiratory symptoms PaO2:FiO2 < 200 mmHg Pulmonary wedge pressure < 18 mmHg RR > 30	Bilateral pulmonary infiltrates on chest imaging
NCT04335071	SpO2 < 93% PaO2 < 65 mmHg Persistent or increasing O2 demand or dyspnea RR ≥ 25	Radiographic evidence compatible with pneumonia
2020-001500-41; EUCTR2020-001500-41-BE	PaO2/FiO2 < 350 while breathing room air in upright position or PaO2/FiO2 < 280 on supplemental oxygen and immediately requiring high flow oxygen device or mechanical ventilation	CXR and/or CT scan showing bilateral infiltrates within last 2 days
NCT04394182	SpO2 < 93% Oxygen therapy escalation (Understanding from less to more need for support: Nasal Cannula; Ventimask ± reservoir) Pa02/Fi02 < 300 mmHg	Worsening of total severity score throughout admission or score at admission > 5 by a diagnostic baseline CT scan
NCT04366232; 2020-001963-10	RR > 30, PaO2 < 90 mmHg ARDS defined by a patient under mechanical ventilation with a PaO2/FiO2 < 300 for > 24 h	No
NCT04357860	Absence of ARDS requiring ONAF or mechanical ventilation	Interstitial pneumonia confirmed by chest radiography or CT
NCT04356690	Intubated or requiring > 4 L/min of supplemental O2 to maintain SpO2 > 92% without intubation	No
NCT04348383	Requiring respiratory support	No
NCT04345445	Dyspnoea OR RR > 20 breaths/min AND O2 sat < 93% on RA OR increasing need for O2 supplementation to maintain O2 sat > 95% on RA	CXR or CT indicative of pneumonia OR worsening findings over time
2020-001255-40; EUCTR2020-001255-40-ES	High oxygen requirements	Evidence of pneumonia
2020-001375-32	Hypoxia	No
NCT04403685	Need for oxygen supplementation to keep SPO2 > 93% or need for mechanical ventilation for less than 24 h	CT with COVID-19 alterations
RPCEC00000311	Need for oxygen therapy to maintain SpO2 > 93% Worsening of lung involvement, defined as one of the following criteria: (a) worsening SpO2 > 3% or decrease in PaO2 > 10%, with FiO2 stable in the last 24 h, (b) need to increase FiO2 in order to maintain a stable SO2 or new need for mechanical installation in the last 24 h, (c) increase in the number and/or extent of consolidation lung areas	Multifocal interstitial pneumonia and worsening of the radiological image
NCT04322773	Need for O2 therapy to maintain SpO2 > 94% or FiO2/PaO2 > 20	Consolidation, ground glass opacities, or bilateral pulmonary infiltration by CT or CXR
NCT04362111	No	No
NCT04423042	No	No
ChiCTR2000030196	No	Severe pneumonia
NCT04339712; 2020-001039-29	No	No
DRKS00021447	No	No
NCT04343963	Dyspnoea Pa:FiO2 < 300 mmHg SpO2 < 90%, or a 3% drop in baseline oximetry, or need to increase supplemental O2 due to chronic hypoxia, as well as the need for supplemental O2	Pneumonia confirmed by imaging studies with increased mortality criteria such as lung infiltrates > 50% of lung fields by CT
2020-001390-76	SpO2 without O2 supplementation < 93% or PaO2/FiO2 < 300 in patients requiring O2 supplementation	Evidence of pulmonary infiltrates at CT or CXR
NCT04377503	Pao2/FIO2 < 200	No
NCT04327505; 2020-001349-37	PaO2/FiO2 < 200 mmHg	No
NCT04359654	SpO2 ≥ 94% on supplementary O2	No
NCT04397497	Requiring O2 supplementation (SpO2 ≤ 92%) and having a PAO2/FIO2 ≤ 300 mmHg	Pneumonia evidenced by CXR or CT with pulmonary infiltrates
NCT04424056	RR > 30/min, PaO2 < 90 mmHg ARDS (mechanically ventilated patient with PaO2/FiO2 < 300 for > 24 h Moderate to severe ARDS (PaO2/FiO2 < 200 to PEEP of at least 8 cmH2O) on invasive mechanical ventilation	COVID-19 pneumonia
NCT04382755	PaO2/FiO2 < 350 or PaO2/FiO2 < 280 on supplemental O2 and immediately requiring Optiflow or mechanical ventilation	CT showing bilateral infiltrates within last 2 days
NCT04330638	PaO2/FiO2 < 350 or PaO2/FiO2 < 280 on supplemental O2 and immediately requiring Optiflow or mechanical ventilation	CXR or CT showing bilateral infiltrates within last 2 days
NCT04324021; 2020-001167-93	PaO2/FiO2 < 300 mmHg and > 200 mm Hg RR ≥ 30 SpO2 < 93%	No
NCT04381052	PaO2/FiO2 < 200, SpO2 < 90% on 4 L, or increasing O2 requirements over 24 h	No
NCT04343989	PaO2/FiO2 < 200, SpO2 < 90% on 4 L, or increasing O2 requirements over 24 h	No
NCT04363502	PaO2/FiO2 < 200, SpO2 < 90% on 4 L, or increasing O2 requirements over 24 h	No
NCT04359290	Recent intubation PaO2/FiO2 ≤ 200 mmHg at a PEEP ≥ 5 mm H2O	CT: pulmonary infiltration
NCT04362813; 2020-001370-30	SpO2 ≤ 93% or PaO2/FiO2 < 300 mmHg	Pneumonia evidenced by CXR or CT with pulmonary infiltrates
NCT04351243	Subject requires high-flow oxygen or meets clinical classification for ARDS	Radiographic evidence of bilateral infiltrates
NCT04517162	SpO2 < 92% or requiring supplemental O2 or mechanical ventilation	Radiologic findings by imaging study: inflammatory infiltrates
NCT04470531	SpO2 < 90% or increasing O2 requirement	Bilateral crackles on auscultation or CXR with bilateral infiltrates
NCT04560205	SpO2 ≤ 93% RR > 30–35	>50% of radiological involvement of lung with typical lesions
NCT04559113	RR > 22	>50% of radiological involvement of lung with typical lesions
NCT04528888	Positive pressure ventilation from >24 h Invasive mechanical ventilation from <96 h PaO2/FiO2 < 150 mmHg	No
NCT04457349	Persistent worsening of respiratory symptoms PaO2/FiO2 < 150 mmHg	No
2020-001645-40	Respiratory distress RR ≥ 30 PaO2/FiO2 < 300 mmHg	Chest imaging confirms lung involvement and inflammation.
2020-001748-24	5 L/min of oxygen to maintain SpO2 at ≥93%	No
NCT04324021; 2020-001167-93	PaO2/FiO2 < 300 mm Hg RR ≥ 30 SpO2 < 93%	COVID-19 pneumonia
NCT04511819	SpO2 ≥ 90% on room air and/or ≥94% on oxygen administration at 2 L/min by nasal cannula	Radiographic evidence of pulmonary involvement consistent with COVID-19

PaO2: partial pressure of O2; SpO2: O2 saturation; Pa:FiO2: partial pressure O2/fraction of inspired O2; Sa:FiO2: O2 saturation measured with pulse oximeter/ fraction of inspired O2; CXR: Chest X-rays; CT: Computerized tomography; RR: respiratory rate; ARDS: acute respiratory distress syndrome.

## References

[1] COVID-19 Treatment Guidelines Panel Coronavirus Disease 2019 (COVID-19) Treatment Guidelines. National Institutes of Health. https://www.covid19treatmentguidelines.nih.gov/.

[2] Huang C., Wang Y., Li X., Ren L., Zhao J., Hu Y., Zhang L., Fan G., Xu J., Gu X. (2020). Clinical features of patients infected with 2019 novel coronavirus in Wuhan, China. Lancet.

[3] Al Sulaiman K.A., Aljuhani O., Eljaaly K., Alharbi A.A., Al Shabasy A.M., Alsaeedi A.S., Al Mutairi M., Badreldin H.A., Al Harbi S.A., Al Haji H.A. (2021). Clinical features and outcomes of critically ill patients with coronavirus disease 2019 (COVID-19): A multicenter cohort study. Int. J. Infect. Dis..

[4] Tisoncik J.R., Korth M.J., Simmons C.P., Farrar J., Martin T.R., Katze M.G. (2021). Into the eye of the cytokine storm. Microbiol. Mol. Biol. Rev..

[5] Shimabukuro-Vornhagen A., Gödel P., Subklewe M., Stemmler H.J., Schlößer H.A., Schlaak M., Kochanek M., Böll B., von Bergwelt-Baildon M.S. (2018). Cytokine release syndrome. J. Immunother. Cancer.

[6] Sinha P., Matthay M.A., Calfee C.S. (2020). Is a “cytokine storm” relevant to COVID-19?. JAMA Intern. Med..

[7] Channappanavar R., Perlman S. (2017). Pathogenic human coronavirus infections: Causes and consequences of cytokine storm and immunopathology. Semin. Immunopathol..

[8] Chatenoud L., Ferran C., Legendre C., Thouard I., Merite S., Reuter A., Kreis H., Franchimont P., Bach J.F. (1990). In vivo cell activation following OKT3 administration. Systemic cytokine release and modulation by corticosteroids. Transplantation.

[9] Gauthier J., Turtle C.J. (2018). Insights into cytokine release syndrome and neurotoxicity after CD19-specific CAR-T cell therapy. Curr. Res. Transl. Med..

[10] Klinger M., Brandl C., Zugmaier G., Hijazi Y., Bargou R.C., Topp M.S., Gökbuget N., Neumann S., Goebeler M., Viardot A. (2012). Immunopharmacologic response of patients with B-lineage acute lymphoblastic leukemia to continuous infusion of T cell–engaging CD19/CD3-bispecific BiTE antibody blinatumomab. Blood J. Am. Soc. Hematol..

[11] Hay K.A., Hanafi L.A., Li D., Gust J., Liles W.C., Wurfel M.M., López J.A., Chen J., Chung D., Harju-Baker S. (2017). Kinetics and biomarkers of severe cytokine release syndrome after CD19 chimeric antigen receptor–modified T-cell therapy. Blood.

[12] Kantarjian H., Stein A., Gökbuget N., Fielding A.K., Schuh A.C., Ribera J.M., Wei A., Dombret H., Foà R., Bassan R. (2017). Blinatumomab versus chemotherapy for advanced acute lymphoblastic leukemia. N. Engl. J. Med..

[13] Mehta P., McAuley D.F., Brown M., Sanchez E., Tattersall R.S., Manson J.J. (2020). COVID-19: Consider cytokine storm syndromes and immunosuppression. Lancet.

[14] Lee D.W., Gardner R., Porter D.L., Louis C.U., Ahmed N., Jensen M., Grupp S.A., Mackall C.L. (2014). Current concepts in the diagnosis and management of cytokine release syndrome. Blood.

[15] England J.T., Abdulla A., Biggs C.M., Lee A., Hay K.A., Hoiland R.L., Wellington C.L., Sekhon M., Jamal S., Shojania K. (2021). Weathering the COVID-19 storm: Lessons from hematologic cytokine syndromes. Blood Rev..

[16] US Department of Health and Human Services (2010). Common Terminology Criteria for Adverse Events (CTCAE).

[17] Davila M.L., Riviere I., Wang X., Bartido S., Park J., Curran K., Chung S.S., Stefanski J., Borquez-Ojeda O., Olszewska M. (2014). Efficacy and toxicity management of 19-28z CAR T cell therapy in B cell acute lymphoblastic leukemia. Sci. Transl. Med..

[18] Neelapu S.S., Tummala S., Kebriaei P., Wierda W., Gutierrez C., Locke F.L., Komanduri K.V., Lin Y., Jain N., Daver N. (2018). Chimeric antigen receptor T-cell therapy-assessment and management of toxicities. Nat. Rev. Clin. Oncol..

[19] US Department of Health and Human Services (2018). Common Terminology Criteria for Adverse Events (CTCAE).

[20] Lee D.W., Santomasso B.D., Locke F.L., Ghobadi A., Turtle C.J., Brudno J.N., Maus M.V., Park J.H., Mead E., Pavletic S. (2019). ASTCT Consensus Grading for Cytokine Release Syndrome and Neurologic Toxicity Associated with Immune Effector Cells. Biol. Blood Marrow Transplant. J. Am. Soc. Blood Marrow Transplant..

[21] Leisman D.E., Ronner L., Pinotti R., Taylor M.D., Sinha P., Calfee C.S., Hirayama A.V., Mastroiani F., Turtle C.J., Harhay M.O. (2020). Cytokine elevation in severe and critical COVID-19: A rapid systematic review, meta-analysis, and comparison with other inflammatory syndromes. Lancet Respir. Med..

[22] Eldanasory O.A., Eljaaly K., Memish Z.A., Al-Tawfiq J.A. (2020). Histamine release theory and roles of antihistamine in the treatment of cytokines storm of COVID-19. Travel Med. Infect. Dis..

[23] Eljaaly K., Alireza K.H., Alshehri S., Al-Tawfiq J.A. (2020). Hydroxychloroquine safety: A meta-analysis of randomized controlled trials. Travel Med. Infect. Dis..

[24] Zhong J., Tang J., Ye C., Dong L. (2020). The immunology of COVID-19: Is immune modulation an option for treatment?. Lancet Rheumatol..

